# Hyperparathyroidism related to an ectopic parathyroid adenoma

**DOI:** 10.11604/pamj.2014.19.135.4931

**Published:** 2014-10-08

**Authors:** Attifi Hicham, Messary Abdelhamid

**Affiliations:** 1 Department of UNT, Military Hospital Moulay Ismail, Meknes, Morocco

**Keywords:** Hyperparathyroidism, adenoma, hypercalcemia

## Image in medicine

A 46-year-old female with a history of muscle weakness, nephrolithiasis and peptic ulcer. She was found to have hypercalcemia (115 mg/l), hypophosphoremia (16 mg/l) and increased PTH serum level (1090 pg/l). In order to discriminate between PH and pseudohyperparathyroidism, a solid-phase, two site chemiluminescent enzyme-labeled immunometric assay directed towards the intact PTH molecule was used and a diagnosis of primary hyperparathyroidism was made. Several studies were performed with the purpose to achieve a pre-operative localization of the oversecreting parathyroid tumor. A cervical sonography showed a normal thyroid gland and no evidence of the tumor was found in the neck. Therefore a 99-TC sesta MIBI was carried out and an accumulation area projected onto the anterior mediastinum was demonstrated (A). Finally a computed tomography scan of the thorax revealed a 4 cm nodule, in the thymic and paratracheal region of the anterior mediastinum (B). The patient underwent surgical resection of the tumor by a lower cervicotomy approach (C,D). Intraoperative parathyroid hormone measurements show a appropriate decrease in PTH levels. A 4x3x2 cm surgical specimen of a parathyroid tissu mass was removed. The anatomopathological examination confirmed the diagnosis of parathyroid adenoma. The post-operative course was uneventful and the patient was discharged in 4th post-operative day with an excellent functional and cosmetic result. A quarterly follow-up was started. PTH serum level and calcemia have been within normal ranges all through 18 months after surgery.

**Figure 1:**
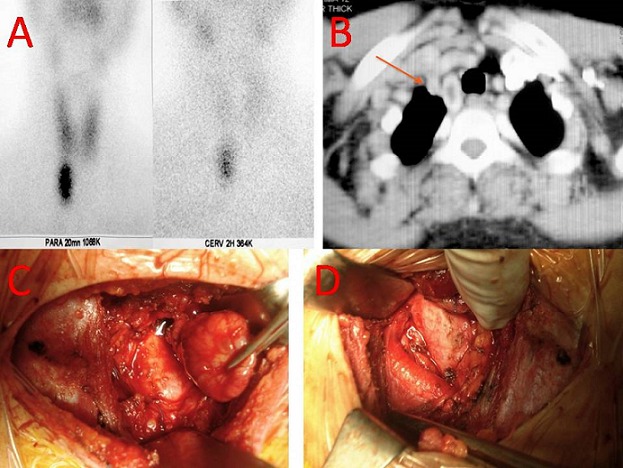
A) a 99-TC sestaMIBI demonstrating an accumulation area projected onto the anterior mediastinum; B) a computed tomography scan of the thorax revealing a 4 cm nodule, in the thymic and paratracheal region of the anterior mediastinum; C) operative view objectifying the resection of the parathyoide adenoma; D) operative view demonstrating the ectopic area of the parathyroid adenoma

